# Identification of Protective CD8 T Cell Responses in a Mouse Model of Zika Virus Infection

**DOI:** 10.3389/fimmu.2019.01678

**Published:** 2019-07-17

**Authors:** Mariah Hassert, Madison G. Harris, James D. Brien, Amelia K. Pinto

**Affiliations:** Department of Molecular Microbiology and Immunology, Saint Louis University, St. Louis, MO, United States

**Keywords:** Zika, dengue, CD8+ T cells, epitope, correlates of protection, immunology and infectious diseases, immunodominance

## Abstract

Many flaviviruses including dengue (DENV), and Zika (ZIKV) have attracted significant attention in the past few years. As many flaviviruses are spread by arthropods, most of the world's population is at risk of encountering a flavivirus, and infection with these viruses has created a significant disease burden worldwide. Vaccination against flaviviruses is thought to be one of the most promising avenues for reducing the disease burden associated with these viruses. The optimism surrounding a vaccine approach is supported by the highly successful vaccines for yellow fever and Japanese encephalitis. Central to the development of new successful vaccines is the understanding of the correlates of protection that will be necessary to engineer into new vaccines. To aid in this endeavor we have directed our efforts to identify correlates of protection that will reduce the disease burden associated with ZIKV and DENV. Within this study we have identified a novel murine ZIKV specific CD8^+^ T cell epitope, and shown that the ZIKV epitope specific CD8^+^ T cell response has a distinct immunodominance hierarchy present during acute infection and is detectible as part of the memory T cell responses. Our studies confirm that ZIKV-specific CD8^+^ T cells are an important correlate of protection for ZIKV and demonstrate that both naïve and ZIKV immune CD8^+^ T cells are sufficient for protection against a lethal ZIKV infection. Overall this study adds to the body of literature demonstrating a role for CD8^+^ T cells in controlling flavivirus infection.

## Introduction

The possibility of becoming infected with an arbovirus has increased dramatically over the past 40 years (WHO). Some of the most prominent emerging arboviruses are members of the *Flaviviridae* family. The *Flavivirus* genus consists of ~70 arthropod-borne viruses with approximately half causing human disease, including Zika virus (ZIKV), West Nile virus (WNV), Dengue virus (DENV), Japanese Encephalitis Virus (JEV), and Yellow fever virus (YFV). The majority of flaviviruses replicate in ticks or mosquitoes and transmit virus to vertebrates by biting. Flaviviruses have also shown their capacity for rapid and explosive spread, as seen in the cases of WNV in 1999 (1), ZIKV in 2015 (2), and YFV in 2016/2017 (3, 4). In all cases, and particularly notable with YFV, diagnosis of the outbreak lagged behind the emergence and spread of the virus. The need for a vaccine to provide protection from emerging flaviviruses is evident and understanding the T cell epitopes responsible for flavivirus protection will aid in identifying the immune protective responses and directly inform vaccine design.

That a ZIKV infection could cause disease was first noted in 1964 (5). Excluding laboratory acquired infections, disease was noted again in febrile children in 1975 (6) and in at least seven patients in Central Java between 1977 and 1978 (7). Prior to the globalization, ZIKV has also been routinely detected by serological assays, when screening for arboviruses in Africa (6, 8–14). In 2017, there were over 1,000 cases of ZIKV disease reported to the CDC in the United States, including US territories. As of September of 2018, that number had dropped to roughly 150; worldwide the numbers have also decreased, likely due to multiple factors including vector control, public awareness and screening, and herd immunity. However, this precipitous drop in ZIKV disease does not mean we are done with ZIKV. While epidemiological surveillance of ZIKV endemic areas is incomplete, they do highlight a common pattern reoccurring disease outbreaks associated with seasonal or environmental changes, similar to what has been seen for DENV and YFV (3, 4). The re-emergence of outbreaks for many pathogens throughout history points to a future where rates of ZIKV infection and disease will be cyclical. Knowing this, we can assume that the incidence of disease associated with ZIKV will re-emerge, and without vaccines or therapeutics to treat infection ZIKV will again become a global health concern.

Diagnosing a ZIKV infection is complicated for a number of reasons, including the high prevalence of asymptomatic infection or generalized symptoms. Indeed, 80–90% of those infected with ZIKV will be asymptomatic or have mild symptoms. A patient presenting with symptoms of acute Zika infection often has generalized symptoms including a mild fever, rashes, and joint pain, which is indicative of a number of infections including DENV(CDC) and the mild symptoms usually resolve within a week and do not often necessitate a visit to a doctor, therefore most ZIKV infections are undiagnosed (15). While the symptoms of disease are relatively short, if present at all, infected individuals can shed virus for several months (16–19) and there are the numerous recent reports demonstrating that ZIKV can be transmitted through contact with bodily fluids (18, 20–22). These factors combined with the evidence that ZIKV infection is linked with congenital malformations and abortions by mother-to-fetus transmission during pregnancy (23–25) makes identifying correlates of protection to design effective treatments and to reduce the risk of disease and viral spread a significant public health priority.

**Figure 1:**
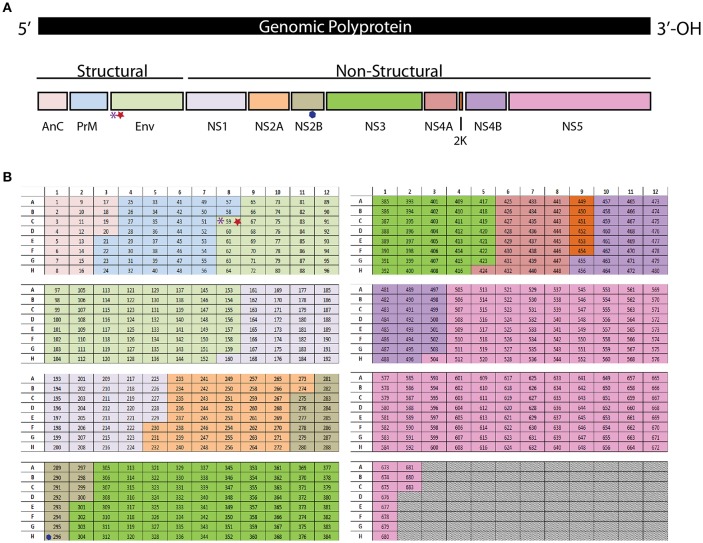
**(A)** The polyprotein encodes three structural (C, prM/M, and E) and seven non-structural (NS1, NS2A, NS2B, NS3, NS4A, NS4B, and NS5) proteins. **(B)** Each peptide from the library is in an individual well in a 96 well plate. The purple asterisk denotes the E294 epitope, The red star denotes the E297 epitope. The blue hexagon denotes the NS2B1478 epitope.

As a members of the family *Flaviviridae*, ZIKV and DENV share many common features in both their structure and genome. ZIKV and DENV are small enveloped viruses that contains a single, positive-sense ~11-kb RNA genome with a 5′ and 3′ untranslated regions flanking a polyprotein (26) (Figure 1A). The polyprotein encodes three structural (C, prM/M, and E) and seven non-structural (NS1, NS2A, NS2B, NS3, NS4A, NS4B, and NS5) proteins (27). The E protein is comprised of three domains (I (E-DI), II (E-DII), and III (E-DIII), with E-DII and E-DIII containing the fusion peptide and putative viral receptor binding site(s), respectively [reviewed in (28, 29)]. Among the structural proteins, prM and E proteins are primary antigenic targets of the humoral immune response in humans for flaviviruses (30–33). As prM and E drive a strong humoral immune response, most vaccines being designed against ZIKV and DENV have tried to incorporate these two key humoral targets.

T cells have been demonstrated to play an important role in protection from a number of flaviviruses by the production of antiviral cytokines and through the killing of infected cells (34–38). In the Ifnar1-/- murine model of DENV infection polyfunctional cytokine producing and cytolytic CD8^+^ T cells prevent uncontrolled replication in peripheral tissues and this protective function can even be elicited through peptide vaccination targeting the immunodominant CD8^+^ T cell epitopes, presenting a point about the importance of eliciting a robust vaccine-mediated CD8^+^ T cell response in protection from DENV (38, 39). This point is also well illustrated in the vaccination of mice with the vaccine strain of YFV (YF-17D), which also elicits a robust CD8^+^ T cell response which has been shown to be important in its vaccine-mediated protection particularly in concert with antibody-mediated protection (37). In the case of neurotropic flaviviruses like WNV and JEV, CD8+ T cells are critical for controlling the infection in neurons and subsequently for protection from disease (40–43). Indeed, it has been shown for both JEV and WNV that vaccination induces robust CD8+ T cell responses and that those cells are an important contribution to the protective capacity of the vaccine (34, 44). Virus-specific and even cross-reactive CD8^+^ T cell peptide epitopes have been identified in mouse models for a number of these viruses and have been an absolutely critical tool in finely dissecting the functions of these cells and the correlates of protection from these viruses (35, 38, 45–47).

Recent studies have identified some human and mouse ZIKV CD4^+^ and CD8^+^ T cell epitopes (35, 48–54) and have begun to identify the role of these T cells in protection against ZIKV. Our group has contributed to the identification of ZIKV specific CD4^+^T cell epitopes in mice noting a novel role for CD4^+^ T cells in the protection against ZIKV neuroinvasive disease in mice (49). In this study we are adding to the current literature for the identification and functional importance of CD8^+^ T cell responses to ZIKV infection, noting that virus-specific CD8^+^ T cells are both necessary and sufficient for survival. We have identified a novel murine ZIKV-specific CD8^+^ T cell epitope, and shown that the ZIKV epitope-specific CD8^+^ T cell response has a distinct immunodominance hierarchy present during acute infection and is detectible as part of the memory T cell responses. These studies confirm the importance of CD8^+^ T cells in ZIKV infection and uniquely noting that naïve CD8^+^ T cells can protect against a lethal viral challenge. As with DENV, understanding the role CD8^+^ T cells will play in protection against severe disease will aid in future vaccine design.

## Materials and Methods

### Ethics Statement

The animal studies were approved by the Saint Louis University Animal Care and Use Committee and done in accordance with the Guide for Care and Use of Laboratory Animals.

### Viruses and Cells

ZIKV (strain PRVABC59) was obtained from BEI (catalog No.: NR-50240) and passaged once in Vero cells (African green monkey kidney epithelial cells) purchased from American Type Culture Collection (ATCC CCL-81). All viruses were titered using a standard focus forming assay (FFA) on Vero cells as previously described (55).

### Mice and Infections

Wild type C57BL/6J and interferon αβ receptor 1 knockout (Ifnar1^−/−^) mice (strain: B6.129S2-Ifnar1^tm1Agt^/Mmjax), commercially purchased from Jackson Laboratories were housed in a pathogen-free mouse facility at the Saint Louis University School of Medicine. For CD8+ T cell depletion studies, 8–12-week-old Ifnar1^−/−^ mice were infected subcutaneously (SC) via footpad injection with 10^5^ FFU of ZIKV. For epitope identification, wild type C57BL/6J mice were infected intravenously (IV) with 10^5^ FFU of virus and boosted 30 days later with 10^5^ FFU of virus. Wild type C57BL/6J mice were used for epitope identification as opposed to Ifnar1-/- mice due to the established persistence of ZIKV that has been observed in Ifnar1-/- mice which we anticipated would impact effective T cell responses due to continuous antigen exposure (49). As we had done previously for the CD4+ T cell studies, (49), for CD8+ T cell adoptive transfer studies, 8–12-week-old Ifnar1^−/−^ mice were infected IV with 10^5^ FFU of ZIKV 1 day after adoptive transfer of cells. During the course of infection mice were assessed for weight loss, signs of neurological disease, and mortality daily. Signs of disease range and in the most severe cases accelerate in the following manner from no apparent disease, limp tail, hind limb weakness, hind limb paralysis, complete paralysis, and death. Occasionally mice will display multiple signs of disease at once, such as limp tail accompanied by hind limb weakness. In such instances, mice are scored as the more severe sign of disease (e.g., hind limb weakness).

### Measurement of Viral Burden

On the indicated days post infection (DPI), intracardiac perfusion (20 ml of PBS) was performed and organs were recovered. EDTA coated tubes were used to collect blood. For organ harvests, the organs were snap frozen and weighed before homogenization with a BeadMill 24 (Fisher scientific). TriReagent RT or RNAzol BD was used to extract viral RNA from the organ lysates or blood, respectively. The following sequences were used to quantify viral RNA by qRT-PCR: Forward- CCGCTGCCCAACACAAG, Reverse- CCACTAACGTTCTTTTGCAGACAT, Probe- AGCCTACCTTGACAAGCAGTCAGACACTCAA.

### Peptide Library

The ZIKV peptide library was constructed using the amino acid sequence from ZIKV-PRVABC59. The library spans the polyprotein and consists of 683 15-mer peptides, overlapping by 10 amino acids. Peptides were reconstituted to 10 mg/ml in 90% DMSO and stored at −80°C. We did not identify any peptides that appeared to be completely insoluble. A final concentration of ~2 μM for each peptide was used for epitope identification. For the peptide stimulation and intracellular cytokine assays the optimal 9-mer peptides (E_294_: IGVSNRDFV, E_297_: SNRDFVEGM, and NS2b_1478_: ICGMNPIAI) were purchase from 21st Century Biochemicals.

### Peptide Stimulation

Splenocytes were harvested from mice 4, 5, or 8 DPI for acute experiments or >30 DPI for assessment of memory responses. Spleens were ground over a 100 μm cell strainer and suspended in RPMI with 10% FBS and HEPES. 10^6^ cells were plated per well in a round-bottom 96-well plate and stimulated with peptide for 6 h at 37°C, 5% CO_2_ in the presence of 10 μg/ml brefeldin A (BFA), and α-CD3 (clone 2C11) was used as a positive control.

### Flow Cytometry

For intracellular cytokine assays, assays were done as described previously (49). Following the peptide stimulation, cells were washed and stained with the cell surface markers: α-CD4-APC-Cy7 (clone RM4-5), α-CD8- PerCP-Cy 5.5 (clone 53–6.7), α-CD3-AF700 (clone 500A2), and α-CD19- AF488 (clone 1D3). After surface staining the cells were fixed, permeabilized, and stained for the intracellular markers: α-IFNγ- APC (clone B27) and α-TNFα- PE (clone Mab11). The cells were analyzed with either an Attune NxT or a BD LSRII.

### Adoptive Transfer of CD8± T Cells

WT C57BL/6J mice (8–10 weeks old) were injected IV with 10^5^ FFU of ZIKV or PBS. Splenocytes were harvested 30 DPI and CD8+ T cells were purified to >97% purity using a Miltenyi negative selection kit. Approximately 3 × 10^6^ cells were administered via IV route to Ifnar1^−/−^ mice 1 day prior to a lethal challenge with ZIKV.

### Statistical Analysis

All statistical analyses were performed using Graph Pad Prism. Statistical differences in survival were determined using a Mantel-Cox test. Differences in disease burden by weight loss were determined using an unpaired *t*-test with Welch's correction. Statistical differences in viral burden were determined by Mann-Whitney test.

## Results

To identify the ZIKV-specific CD4+ (49) and CD8+ T cell responses we have used a peptide library screening method. The peptide library was generated from the amino acid sequence of the ZIKV strain PRVABC59 (Accession #U501215.1). The peptide library is comprised of 683 peptides, each peptide is 15 amino acids (aa) long and the peptide sequences overlap by 10aa. Each peptide from the library is in an individual well in a 96 well plate (Figure 1B). The resulting peptide library is spread across eight plates. The peptides are reconstituted in 90% DMSO to make a stock of peptides at 10 mg/ml which was used for all the studies detailed below.

**Figure 2:**
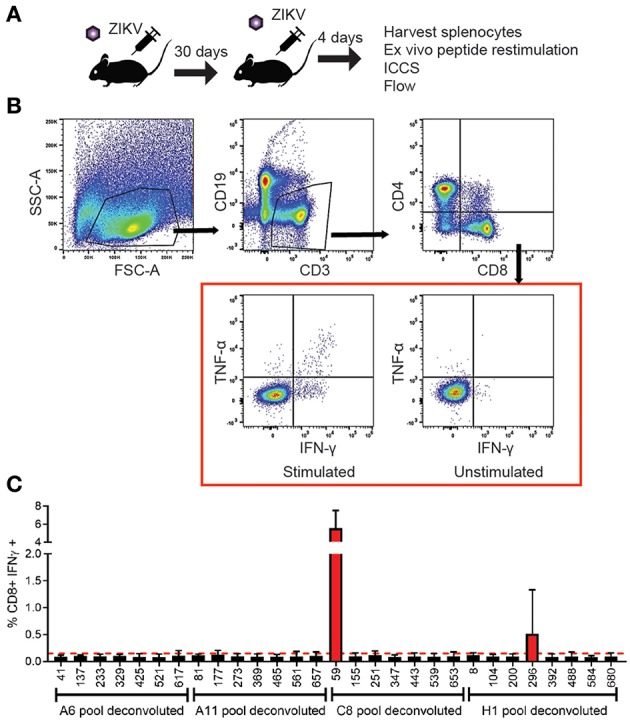
Identification and functional analysis of ZIKV-specific CD8+ T cell epitopes. **(A)** Diagram of C57BL/6J mice infected 10^5^FFU of ZIKV IV, rested for 30 days then boosted with a second ZIKV infection again with 10^5^ FFU of virus IV. Spleens were harvested 4 days after the boost. The splenocytes isolated from the ZIKV boosted animals were then used in the intracellular cytokine assay. Peptide pools were then used to identify the antigen experienced CD8^+^ T cells using an intracellular cytokine assay. **(B)** Representative gating strategy used to analyze intracellularly with antibodies specific for the mouse cytokines interferon-γ (IFN-γ) and tumor necrosis factor-α (TNF-α). Splenocytes were harvested and stimulated with peptide pools in the presence of brefeldin A. Cells were stained for surface markers (CD3, CD19, CD4, and CD8), stained intracellularly for IFNγ and TNFα and analyzed by flow cytometry. Cells were gated using a lymphocyte gate, CD19^−^, CD4^−^, CD8^+^, and were functionally analyzed by expression of IFNγ. Data is presented as the percent of CD8+ T cells that produced IFNγ in response to pooled peptide stimulation. **(C)** Peptide pools from wells C8 and H11 from the individual were deconvoluted and the cytokine responses detected from the individual 15mer peptides from the library allowed us to identify 2 possible CD8^+^ T cell epitopes from the deconvoluted peptide pools. The 15mer peptide epitope was considered positive if the results were two times over background unstimulated samples. The 15mer peptide found in well 59 of the peptide library mapped to and area overlapping between PrM and E was identified from the C8 pool, and a 15mer peptide found in well 196 of the peptide library mapped to NS2b was identified from the H1 pool.

To identify the antigen specific CD8+ T cells in our primary screen we infected wild type C57BL/6 mice with 10^5^ FFU of virus IV. It was confirmed that the dose and route of virus infection would result in transient replication sufficient for effective antigen presentation by harvesting the spleen and lymph nodes of a subset of these mice on days 3 and 6 post infection and virus detection via qPCR (Supplementary Figure 1). The mice were then rested for at least 30 days then boosted with a second ZIKV infection again with 10^5^ FFU of virus IV to allow for the most robust response driven by anamnestic recall (Figure 2A). The splenocytes were isolated 4 days after the boost and were plated into 96 well plates and stimulated for 6 h with Brefeldin A (BFA) and a pool of 6–8 peptides. To generate these pools we combined the same well from each of the eight plates from the aliquoted library shown in Figure 1B. Unstimulated cells were setup as a negative control and as a positive control, anti-CD3 (45-2C11) was used to stimulate antigen experienced CD8^+^ T cells from the ZIKV boosted animals. After the stimulation, splenocytes were stained with the cell surface antibodies, α-CD3, α-CD8, α-CD4, and α-CD19 then stained intracellularly with antibodies against the mouse cytokines interferon-γ (IFN-γ) and tumor necrosis factor-α (TNF-α) (Figure 2B). The cytokine responses detected from the pooled peptide wells allowed us to identify 2 possible CD8^+^ T cell epitopes within the peptide pools. We detected responses to peptides pools located in wells C8 and H1 in our original screen. The individual peptides within the peptide pools were identified by repeating the ZIKV boosting strategy in the C57BL/6J mice. The individual 15mer peptides that induced cytokine responses in our ZIKV boosted CD8+ T cells were identified by expanding the positive pooled wells (Figure 2C). With this approach we identified two wells that contained peptides which induced a cytokine response suggesting that we had one epitope in well 59 and one epitope in well 296. For both of these wells the amount of IFN-γ produced following peptide stimulation was at least two times higher than the background unstimulated cell control well. Shown in Figure 2C, the peptide identified in well 59 is the most dominant CD8+ T cell epitope we identified. The epitope in well 59 mapped to the a very proximal region of the Envelope protein and the epitope in well 296 mapped to the NS2b protein (Figures 1A,B).

**Figure 3:**
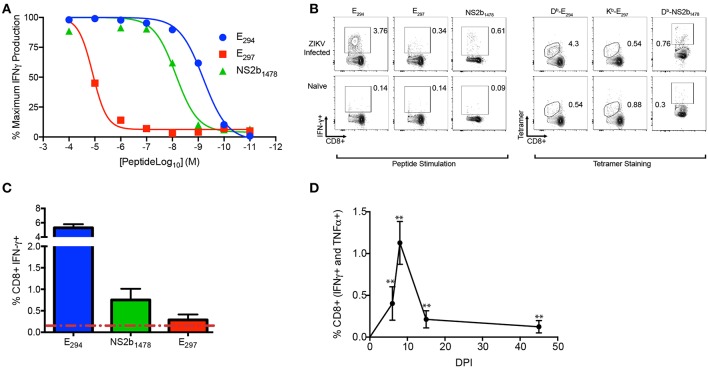
ZIKV-specific CD8+ T cell epitope identification in the acute and memory phases of infection. **(A–D)** C57BL/6J mice (*n* between 3 and 5) were injected IV with 10^5^ FFU of ZIKV bled or harvested at the timepoints indicated **(A)** The functional avidity of the CD8 T cell epitopes identified. On day 8 post infection splenocytes were harvested and stimulated with the indicated peptides (E_294_, E_297_, and NS2b_1478_) with the concentrations listed on the y-axis in the presence of brefeldin A. Cells were gated using a lymphocyte gate, CD19^−^, CD4^−^, CD8^+^, and were functionally analyzed by expression of IFNγ. Data is presented as the normalized maximal percent of CD8+ T cells that produced IFNγ in response to stimulation). **(B)** Representative flow plot comparing tetramer binding frequency to IFN-γ response for E_294_, E_297_, and NS2b_1478_. On day 8 post infection splenocytes were harvested and either stimulated with the E_294_, and E_297_ peptides or stained with peptide specific tetramers. **(C)** Expression hierarchy of E_294_, NS2B_1478_, and E_297_ during the acute infection. On day 8 post infection splenocytes were stimulated with E_294_, NS2B_1478_, or E_297_ peptides in the presence of BFA and the proportion of the CD8+ T cell response dedicated to each of epitope was determined. **(D)** Functional response to E_294_ is preserved in memory. C57BL/6J mice were injected IV with 10^5^ FFU of ZIKV and on day 0, 5, 8 15, and 45 mice were bled and were stimulated and stained as described above. Data is presented as the percent of CD8+ T cells that produced IFNγ and TNFα in response to stimulation. Data is from a single experiment (*n* = 6). Asterisks indicate values that are statistically significant (^**^p < 0.005) as determined by Mann-Whitney test.

Using the information from the 15mers in the peptide screen we set out to identify the optimal peptide sequence for each of the epitopes (Table 1). We named the ZIKV peptide epitopes using the same nomenclature as used previously for WNV (46), with the abbreviated name of the viral protein followed by the number of the amino acid based upon the flavivirus open reading frame, for example E_294_ would mean the epitope began at the 294th amino acid in the open reading frame and is present in the E protein. For well 59 we were surprised to discover that the sequence analysis suggested that there were two possible epitopes within this region E_294_ and E_297_ which were determined to be a H2-D^b^ and H2-K^b^ epitopes, respectively. Analysis of the literature confirmed this observation that there were two epitopes identified within this region (56). The NS2b epitope in well 296 was identified as NS2B_1478_. To determine the avidity of the T cell responses to each identified epitope we performed peptide dose response assays on day eight after intravenous (IV) ZIKV infection (10^5^ FFU) (Figure 3A). E_294_ and NS2b_1478_ both had similar T cell peptide functional avidities with Log_EC50_ of −9.2 for E_294_ and Log_EC50_ of −8.4 for NS2b_1478_, which is line with other identified T cell epitopes. For E_297_ the T cell response dropped off rapidly, with T cell peptide functional avidities with Log_EC50_ of −4.8 suggesting a much lower functional avidity than the two other epitopes identified in the screen. For E_294_, E_297_, and NS2b_1478_, we also had tetramers made by the NIH Tetramer facility. We then used splenocytes from day eight ZIKV infected C57BL/6J mice to determine the tetramer binding frequency compared to the intracellular cytokine IFN-γ response at the same time point (Figure 3B). For E_294_ and NS2b_1478_, the tetramer analysis demonstrated a similar percentage and number of responding CD8+ T cells as we had seen with the intracellular cytokine staining for IFN-γ. This finding emphasizes validity of our cytokine-based screening approach and the likelihood that we have identified the optimal 9-mer peptide sequence for these two epitopes. However, we were unable to detect any staining with the E_297_ tetramer although we did see responses above background by intracellular cytokine staining. We are continuing to investigate this observation and our current interpretation is that we have not yet identified the optimal E_297_ epitope.

**Table 1:** Epitope identities.

**Name**	**Amino acid sequence**
E_294_	IGVSNRDFV
E_297_	SNRDFVEGM
NS4b_1478_	ICGMNPIAI

To establish the expression hierarchy for the peptide epitopes we identified, C57BL/6J mice were infected with 1 × 10^5^ FFU of ZIKV IV and after 8 days the mice were sacrificed and splenocytes isolated. The individual peptides were used to stimulate the splenocytes in the presence of BFA for 6 h then cells are stained with cell surface and intracellular cytokines antibodies to identify the responding antigen specific CD8+ T cells (Figure 3C). The immunodominance hierarchy was determined by identifying the proportion of the acute CD8+ T cell response dedicated to each of the ZIKV-specific epitopes. The expression hierarchy of the immunodominant epitopes was determined to be E_294_, NS2B_1478_, and E_297_. We next wanted to demonstrate that the E_294_ epitope specific CD8+ T cell response was preserved into memory. We repeated our infection of C57BL/6J mice with 1 × 10^5^ FFU of ZIKV IV bleeding sequentially on days 5, 8, 14, and 45 DPI, performing an intracellular cytokine stain for each time point (Figure 3D). An E_294_ polyfunctional response was detectable in all mice out to day 45 suggesting that the ZIKV specific CD8+ T cell response is preserved. Similarly, NS2B_1478_ polyfunctional memory responses were also detected in splenocytes from ZIKV infected C57BL/6J mice at least 45 days post infection (Supplementary Figure 2A). While we were able to observe a polyfunctional memory response in splenocytes stimulated with E_297_ from ZIKV infected C57BL/6J mice at least 45 days post infection relative to naïve animals (Supplementary Figure 2B), we were unable to show that these responses were statistically significantly different due to limited n.

**Figure 4:**
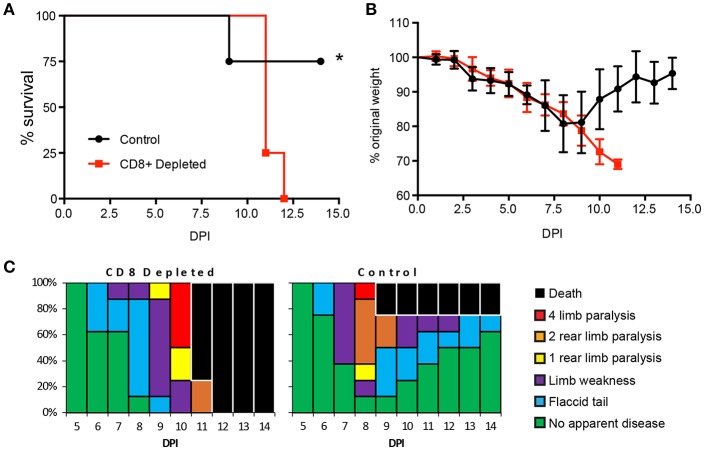
CD8+ T cells are necessary for protection from ZIKV challenge. **(A)** Survival of 10–12-week-old Ifnar1^−/−^ mice following CD8+ T cell depletion and inoculation with 10^5^ FFU of ZIKV by footpad injection (*n* = 9 control, *n* = 12 depleted). On day −3 and day 0, mice were administered 100 μg of depleting antibody anti-CD8 or isotype control intraperitoneally (*n* = 9 or 12 mice per group, respectively). Survival differences were statistically significant (^*^p = 0.018) as determined using a Mantel-Cox test. **(B)** Weight loss during acute ZIKV infection of 10–12-week-old mice. As a measure of disease, mice were weighed daily for 15 days. Prior to depleted mice succumbing to infection there were no significant differences between the CD8^+^ depleted group and control group as determined using an unpaired *t*-test with Welch's correction. **(C)** Clinical scoring associated with acute ZIKV infection. Mice were evaluated for signs of neurological disease daily and graphed on each day as a percentage of mice displaying that disease indicator. Signs of disease range and in the most severe cases accelerate in the following manner from no apparent disease, limp tail, hind limb weakness, hind limb paralysis, complete paralysis and death. All data is a compellation of 2 independent experiments.

Previous studies have established an important role for CD8+ T cells in protection against a lethal ZIKV challenge in type 1 interferon insufficient mice (35, 54, 56–58). To confirm the role of CD8+ T cells in our hands we depleted CD8+ T cell from 8–12-week-old type I interferon receptor deficient (Ifnar1^−/−^) mice, which are the same MHC haplotype (H2-b) as the C57BL/6 mice used in our epitope mapping experiments (Figure 4A). The Ifnar1^−/−^ mice received the CD8+ T cell depleting antibody 3 days prior to infection and a second dose on the day of subcutaneous (SC) infection with 1 × 10^5^ focus forming units (FFU) of ZIKV. Following ZIKV infection we monitored the mice daily, recording: mortality, weight, and clinical signs of disease (Figures 4A–C). In both the CD8 depleted and control mice we saw evidence of ZIKV infection and disease, which included weight loss, and temporary hind limb paralysis. There was a significant difference in the mortality between the CD8 depleted and control mice, as 100% of the CD8 depleted mice succumb to ZIKV, compared to a 25% mortality of the Ifnar1^−/−^ control mice (Figure 4A). Unlike what we had previously observed with CD4^+^T cell depletions (49), we did not observe any differences in weight loss between the control and depleted mice prior to the mice succumbing to infection (Figure 4B) and the disease scores between the two groups were similar with the onset of disease occurring 6 days post infection (Figure 4C). The results of these studies confirmed the necessity of CD8+ T cells for the control of ZIKV infection in Ifnar1^−/−^ mice, but point to a different role for CD8+ T cells in the control of ZIKV as compared to what we had previously observed with CD4^+^T cells (49).

**Figure 5:**
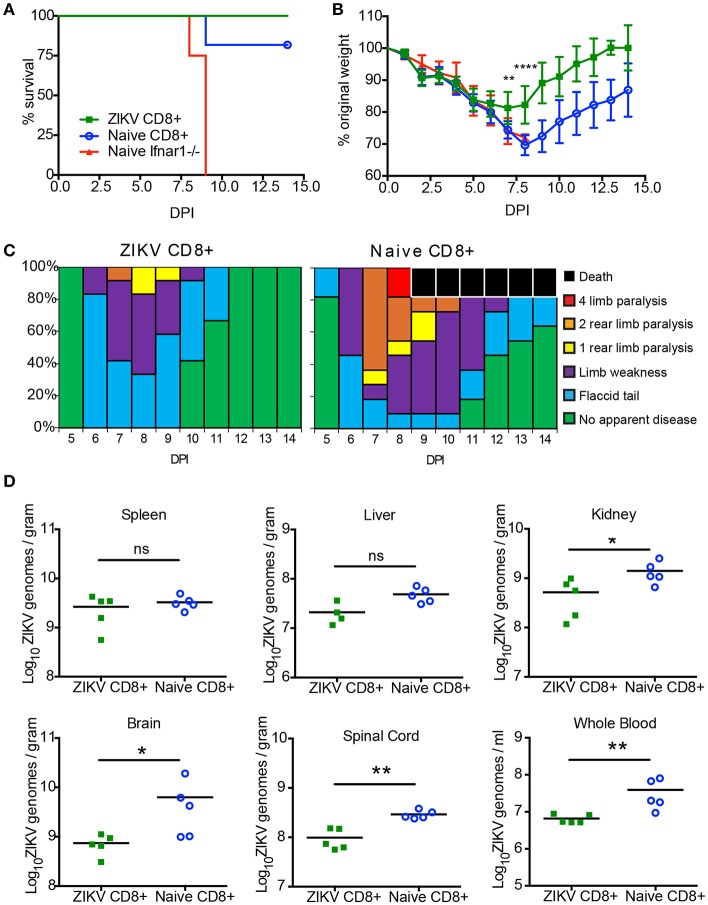
CD8+ T cells are sufficient to protect against a lethal ZIKV challenge. **(A)** Survival of 10–12-week-old mice following adoptive transfer of CD8+ T cells and IV route ZIKV challenge. At 30 days post infection, CD8+ T cells were isolated to >97% purity from ZIKV infected or naïve C57BL/6J mice and transferred IV into 10- to 12-week-old Ifnar1^−/−^ mice (~3 × 10^6^ /mouse) 1 day prior to IV infection with 10^5^ FFU of ZIKV (*n* = 9–11 per group). Survival differences were not statistically significant between the two groups that received CD8 T cells but were statistically different than mice that received PBS as determined by Mantel-Cox test. **(B)** Weight loss during IV ZIKV infection of 10–12-week-old mice following adoptive transfer. As a measure of disease, mice were weighed daily for 14 days. There were significant differences between the mice that receive ZIKV immune CD8^+^ T cells from group compared to the mice that received naïve CD8 T cells on day 7 (^**^p = 0.001) and day 8 (^****^p < 0.0001) determined using an unpaired *t*-test with Welch's correction. **(C)** The clinical scores associated with IV ZIKV challenge following adoptive transfer. Mice were evaluated for signs of neurological disease daily and graphed on each day as a percentage of mice displaying that disease indicator. Signs of disease range and in the most severe cases accelerate in the following manner from no apparent disease, limp tail, hind limb weakness, hind limb paralysis, complete paralysis, and death. **(D)** Viral burden in the peripheral and CNS tissues after CD8^+^ adoptive transfer and ZIKV infection. Ifnar1-/- mice that received CD8^+^ T cells from naïve or day 30 ZIKV immune C57BL/6J Ly5.1 mice were infected with 10^5^ FFU ZIKV via IV route. On day 14 (*n* = 5 per group) post-infection, organs were harvested, snap frozen, weighed, and homogenized. Levels of viral RNA were quantified by qPCR in whole blood, liver, spleen, kidney, spinal cord, and brain. Data are shown as Log_10_ focus-forming unit equivalents (eq.) (as determined by standard curve) per gram or ml of tissue or blood, respectively. Asterisks indicate values that are statistically significant (^*^p < 0.05, ^**^p < 0.001) as determined by Mann-Whitney test.

After demonstrating the necessity of CD8+ T cells for protection from ZIKV in Ifnar1-/- mice, we next examined if CD8+ T cells from a ZIKV immune mouse were sufficient for protection. We isolated CD8+ T cells from naïve or ZIKV immune C57BL/6J Ly5.1 mice. The Ly5.1. mice were infected with ZIKV 30 days prior to adoptive transfer. On the day of the transfer, spleens were harvested from naïve or ZIKV immune Ly5.1. mice and CD8+ T cells were isolated by negative selection with magnetic beads. The CD8+ T cell isolation resulted in a purity of approximately 97% as determined by flow cytometry (data not shown). 4 × 10^6^ naïve or ZIKV CD8+ T cells were then adoptively transferred into 8-week-old Ifnar1^−/−^ mice 1 day prior to 10^5^ FFU ZIKV IV lethal challenge. The mortality, weight loss and clinical scores of the ZIKV infected Ifnar1^−/−^ mice were monitored for 14 days (Figures 5A–C). All of the Ifnar1^−/−^ mice that received the CD8+ T cells from the ZIKV immunized mouse survived, and surprisingly 80% of the mice that received the naive CD8+ T cells survived the lethal challenge (Figure 5A). The mice that received the CD8+ T cells from the ZIKV immune mice lost significantly less weight than the mice that received the naïve T cells on days 8–14 (Figure 5B). The ZIKV infected Ifnar1^−/−^ mice that received naïve T cells had an earlier detection of clinical scores than the mice that receive the ZIKV immune CD8+ T cells, day 5 compared to day 6, and the duration of detectible clinical scores in the naïve mice last longer with 20% of the mice still showing evidence of disease at day 14 post infection (Figure 5C). While all mice that received the adoptively transferred CD8+ T cells showed some signs of diseases as detected by the clinical scores the proportion of mice with elevated disease scores also was higher in the Ifnar1^−/−^ mice that received the naïve CD8+ T cells with as many as 60% of the mice showing signs of 2 rear limb paralysis on day 7 as compared to 10% in the mice that had received the ZIKV immune CD8+ T cells.

Finally, we were interested to see if the CD8+ T cells transferred from the ZIKV immune mice were better able to prevent ZIKV persistence compared the transferred naïve CD8+ T cells (Figure 5D). We have previously shown that ZIKV could persist in Ifnar1^−/−^ mice (49). To determine if the transferred CD8+ T cells from the ZIKV immune mice could clear the ZIKV infection we harvested organs from five of the remaining ZIKV infected mice that received the naïve and ZIKV immune CD8+ T cells 14 days post infection. We titered the virus in the spleens, livers, kidneys, brains, spinal cords, and from whole blood using quantitative real-time PCR. For all mice in both groups we were able to detect virus in all the organs analyzed suggesting that the ZIKV immune CD8+ T cells alone were not able to clear virus from the Ifnar1^−/−^ mice. However, in multiple organs, (kidney, brain, and spinal cord as well as whole blood) we did note a significantly lower viral titer in the mice that had received CD8+ T cells from ZIKV immune mice compared to the mice that had received naïve CD8+ T cells, suggesting that the virus specific T cells were better at controlling the virus in the Ifnar1^−/−^ mice.

The results of our study suggest that there is a strong detectible CD8+ T cell response to ZIKV following infection and that ZIKV-specific CD8+ T cells are able to reduce the signs of disease and protect against a lethal ZIKV infection. Surprisingly, we saw a similar level of protection against mortality when we adoptively transferred naïve CD8+ T cells into the lethally challenged mice. The mechanisms responsible for this protection are currently being investigated. These results indicate that ZIKV specific CD8+ T cells are both necessary and sufficient to protect against lethal ZIKV infection.

## Discussion

Achieving a protective humoral immune response has driven much of the focus for effective vaccine design. However, we now understand that most of the highly effective vaccines incorporate both humoral and T cell responses. For flaviviruses, T cell responses in particular have proven to be highly relevant for controlling viral infection and reducing disease severity (35, 59–63). Yellow fever virus vaccine (YFV-17D) provides one of the best examples for the need to consider the T cell response for the development of an effective vaccine [reviewed in (64)]. More recently studies have demonstrated that strong T cell responses is important for controlling DENV infections and for reducing the possible effects of antibody dependent enhancement (ADE) (65, 66). These studies point to the important role for the cellular immune responses during flavivirus infection and vaccine-mediated protection and begin to highlight the shift in focus for vaccine development to designing vaccines that stimulate both the cellular and humoral immune responses. There are multiple requirements for the development of a vaccine that incorporates the cellular immune response including (1) the identification of immune-stimulatory antigenic epitopes of the virus, (2) confirmation of the protective capacity of the cellular response, and (3) evidence that the epitope specific T cell responses are maintained into memory. While ultimately these studies must be carried out with human vaccine trials, animal models have been critical in establishing the correlates of protection that should be incorporated into informing an effective vaccine strategy. Epitope identification has long been established as the fundamental foundational work that needs to be done to begin to understand the immune correlates of T cell protection. ZIKV-specific CD8+ T cell epitope identification using animal models allows for the initial study of immune correlates of protection and opens up new models assessing vaccine efficacy.

We set out to identify H2-K^b^-and H2-D^b^ restricted ZIKV CD8+ T cell epitopes using the production of intracellular cytokine responses following peptide stimulation from a full length overlapping peptide library (Figure 1). Traditionally the production of IFN-y and TNF-α has been correlated with potent effector function with flaviviruses (67), so we hypothesized that CD8^+^ T cell responses we identified using this approach would correlate with potent protective responses. We show here that ZIKV mounts a potent CD8+ T cell response in mice that persists into memory. Using a whole genome peptide library approach, we were able to identify three MHCI-restricted CD8+ T cell epitopes; two corresponding to the Envelope (E_294_) (E_297_) and one corresponding to the non-structural protein NS2b (NS2b_1478_) (Figure 3). Notably this is the first reported study that identified NS2b_1478_ as a ZIKV-specific epitope in mice on a C57BL/6J background. Using this approach, we were not able to detect responses to some of the previously identified CD8+ T cell epitopes from C57BL/6J mice (35). This may be due to our use of IFN-y and TNF-α as a means to identify ZIKV-specific CD8+ T cell epitopes or possibly the use of a full15-mer peptide library as opposed to using epitope prediction software. By requiring the T cells to produce cytokine as a means of identification we biased our results toward these effector cell populations potentially missing ZIKV specific CD8+ T cell populations that do not make these cytokine responses. As such, it should be noted that studies have demonstrated that the T cells that don't produce IFN-y may be important for control of some viruses (68). Additionally, the use of 15mer amino acids to screen for epitopes requires some level of processing to the optimal 9mer for CD8+ T cell stimulation and identification, therefore we could miss epitopes that could not be optimally processed. To gain the most accurate picture of the ZIKV-specific CD8+ T cell response we and others will need to conduct further studies.

It is worth noting that in our initial prime-boost based screening assays, we did not detect strong ZIKV-specific CD8+ T cell responses in the Ifnar1^−/−^ mice compared to the immune competent C57BL/6J (data not shown). Because we have previously demonstrated Ifnar1^−/−^ mice had persistent viral titers for >30 days after infection with ZIKV (49), we hypothesized that the virus specific T cell population was more exhausted in the Ifnar1^−/−^ mice due to persistent antigenic stimulation. As we relied on a functional assay with cytokine production to map the T cell response in our animals we used C57BL6/J mice, which are of the same MHC haplotype as Ifnar1-/- mice (H2-b). Based on the epitopes we identified in this study and the CD4+ T cell epitopes we identified previously (49) we are currently conducting studies using CD4+ and CD8+ T cell tetramers to further investigate this observation of potential exhaustion.

After the identification of the virus specific CD8+ T cell responses in our model we next wanted to determine if CD8+ T cells were necessary for protection against a sublethal ZIKV challenge. Through depletion studies we demonstrated that the loss of CD8+ T cells lead to a significantly higher mortality in susceptible Ifnar1-/- mice as compared to mice that had received an isotype control antibody (Figure 4A). However, unlike what we had observed for the CD4+ T cell depletion studies, we noted that there were no significant differences in weight loss between the CD8+ depleted and control mice prior to the depleted mice succumbing to infection (Figure 4B). Based on our clinical scoring observations the onset of disease was similar between the groups with some mice in both groups showing signs of a flaccid tail on day 6 post infection (Figure 4C). Notably more of the isotype control mice showed signs of neurological disease and limb paralysis earlier (Day 8) than the CD8+ depleted mice (Day 9). These results are in line with the previously published observations by Jurado et al that suggest that CD8+ T cells may cause some of the neuropathology seen in ZIKV disease in mice (57). However, ultimately all of the CD8+ T cell depleted mice succumb to infection where the most of the isotype control treated mice recovered demonstrating that CD8 T cells are necessary for protection against ZIKV mortality in Ifnar1-/- mice.

Multiple studies have suggested a dominant role of CD8+ T cells in controlling ZIKV infection (35, 50, 51, 56, 57, 69–71) although at least one study highlights the potential for ZIKV specific CD8+ T cells to play an immunopathological role in neuroinvasive disease (19). We sought to determine if ZIKV specific CD8+ T cells were sufficient for protection against a lethal ZIKV challenge in our model (Figure 5). Similar to what has previously been observed we noted that CD8+ T cells transferred from ZIKV immune mice were protective against a lethal ZIKV challenge. However, we also observed a similar level of protection from mortality when we adoptively transferred naïve C57BL6/J CD8+ T cells into the Ifnar1-/- mice prior to lethal challenge. The mice that received the naïve CD8+ T cells did lose significantly more weight than the mice that received CD8+ T cells from ZIKV immune mice. Additionally, 75% of the mice that received naïve T cells showed some level of limb paralysis as compared to 20% of the mice that received CD8+ T cells from immune mice. Moreover, mice that received CD8+ T cells from ZIKV immune mice had reduced viral burden in multiple organs. Taken together, these results indicate that CD8+ T cells from ZIKV immune mice were sufficient to reduce the viral burden, morbidity, and clinical signs of ZIKV disease, while both naïve and ZIKV immune CD8+ T cells from C57BL/6J mice were sufficient to protect Ifnar1-/- mice from a lethal challenge relative to un-manipulated infected Ifnar1-/- mice.

In summary, our current study characterizes the protective capacity of CD8+ T cells during a ZIKV infection in a susceptible mouse model. We confirmed the identification of the ZIKV specific epitopes E_294_ and E_297_ and identified a novel ZIKV CD8+ T cell epitope NS2B_1478_. In this study we also demonstrated that CD8+ T cells were necessary for protection against ZIKV lethality and that while CD8+ T cells from ZIKV immune C57BL/6J mice contributed to reduced viral burden and ZIKV induced morbidity, both naïve or T cells from ZIKV immune mice were sufficient for protection from lethality. These results highlight a need for further studies looking into the role of the virus specific CD8+ T cells directed against our identified epitopes in protection from ZIKV infection.

## Data Availability

All datasets generated for this study are included in the manuscript and/or the Supplementary Files.

## Ethics Statement

This study was carried out in accordance with the recommendations of Guide for Care and Use of Laboratory Animals of the National Institutes of Health. The protocol was approved by the Saint Louis University Animal Care and Use Committee.

## Author Contributions

MH and MGH performed the experiments and contributed to the manuscript. JB and AP wrote the manuscript and directed the research.

### Conflict of Interest Statement

The authors declare that the research was conducted in the absence of any commercial or financial relationships that could be construed as a potential conflict of interest.

## Supplementary Material

Supplemental Figure 1 qPCR to measure viral load in lymphoid organs of mice used in epitope mapping. Wild type C57BL/6J mice were infected with 10^5^ FFU of ZIKV via IV route. On days 3 and 6 post infection, the spleens and lymph nodes were harvested, weighed, and homogenized. qRT-PCR was used to quantify the viral load in each organ.

Supplemental Figure 2 *In vivo* expansion of NS2b_1478_ and E_297_ specific cells. Wild type C57BL/6J mice were infected with 10^5^ FFU of ZIKV via IV route. At days 0, 5, 8, or 45 post-infection, splenocytes were harvested and stimulated with NS2b_1478_ peptide **(A)** or E_297_ peptide **(B)** for 6 h in the presence of brefeldin A. Cells were stained for surface markers (CD3, CD19, CD4, and CD8), stained intracellularly for IFNγ and TNFα and analyzed by flow cytometry. Cells were gated using a lymphocyte gate, CD19^−^, CD4^−^, CD8^+^, and were functionally analyzed by expression of IFNγ and TNFα. Data is presented as the percent of CD8+ T cells that produced both IFNγ and TNFα in response to peptide stimulation. Asterisks indicate values that are statistically significant (^*^*p* < 0.05) as determined by Mann-Whitney test.
