# Supplementary figures and images for: Identification of Protective CD8 T Cell Responses in a Mouse Model of Zika Virus Infection
Source: Front Immunol. 2019 Jul 17;10:1678. doi: 10.3389/fimmu.2019.01678 (PMC6652237; doi:10.3389/fimmu.2019.01678)

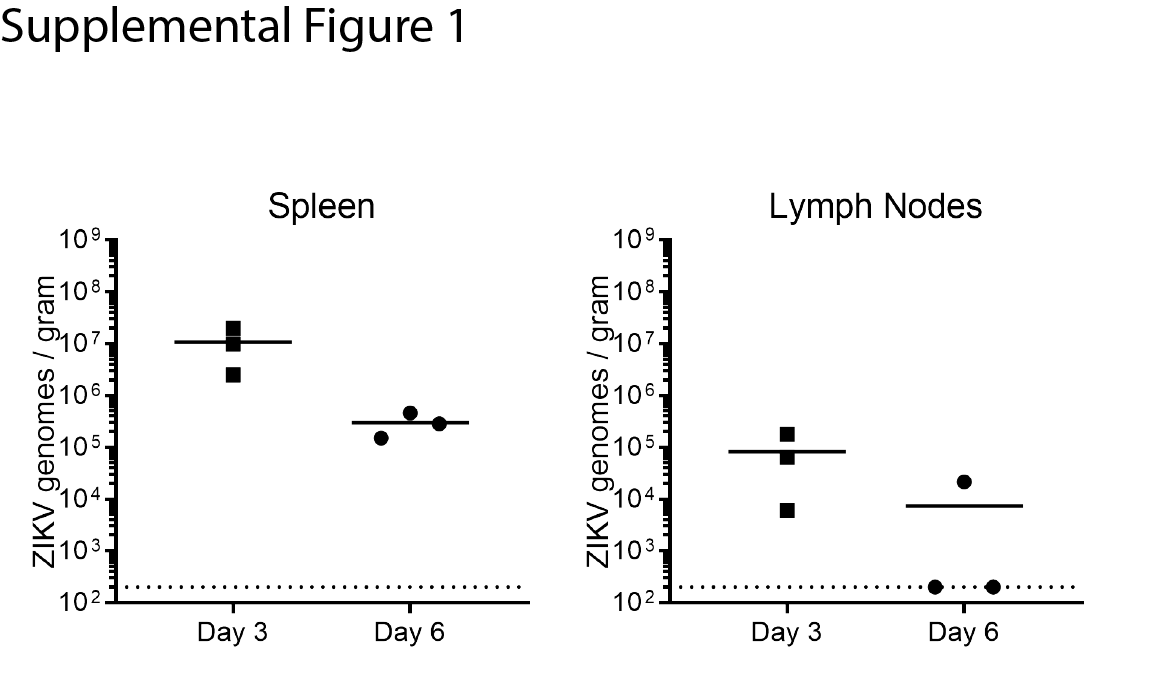

Supplement: Supplemental Figure 1 — qPCR to measure viral load in lymphoid organs of mice used in epitope mapping. Wild type C57BL/6J mice were infected with 105 FFU of ZIKV via IV route. On days 3 and 6 post infection, the spleens and lymph nodes were harvested, weighed, and homogenized. qRT-PCR was used to quantify the viral load in each organ. [file Image_1.TIF]

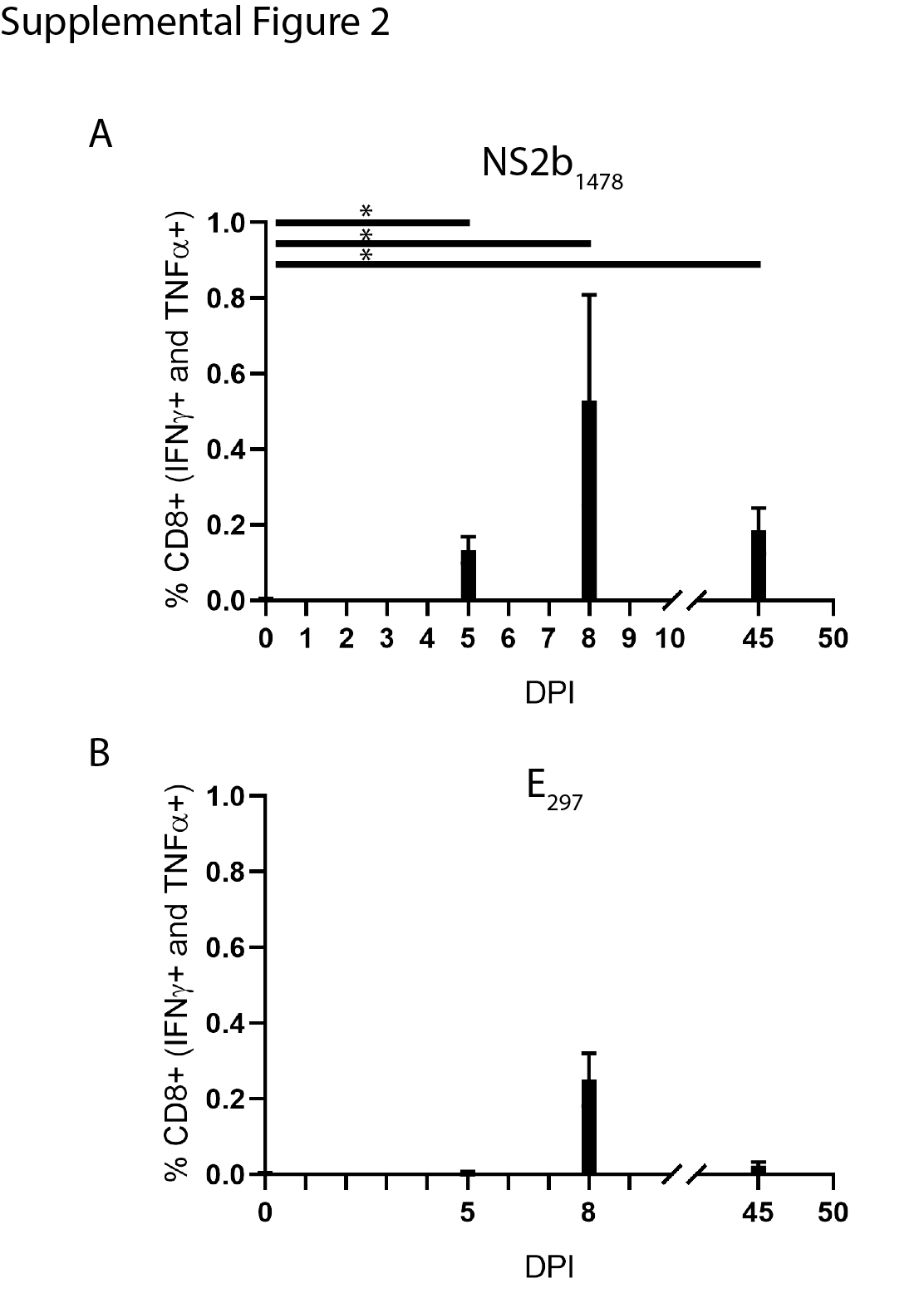

Supplement: Supplemental Figure 2 — In vivo expansion of NS2b1478 and E297 specific cells. Wild type C57BL/6J mice were infected with 105 FFU of ZIKV via IV route. At days 0, 5, 8, or 45 post-infection, splenocytes were harvested and stimulated with NS2b1478 peptide (A) or E297 peptide (B) for 6 h in the presence of brefeldin A. Cells were stained for surface markers (CD3, CD19, CD4, and CD8), stained intracellularly for IFNγ and TNFα and analyzed by flow cytometry. Cells were gated using a lymphocyte gate, CD19−, CD4−, CD8+, and were functionally analyzed by expression of IFNγ and TNFα. Data is presented as the percent of CD8+ T cells that produced both IFNγ and TNFα in response to peptide stimulation. Asterisks indicate values that are statistically significant (*p < 0.05) as determined by Mann-Whitney test. [file Image_2.TIF]
